# Supplementary figures and images for: Neurodevelopmental outcomes in preschool children with congenital heart defects: A case-control study using Ages & Stages Questionnaire
Source: PLoS One. 2026 Mar 25;21(3):e0341135. doi: 10.1371/journal.pone.0341135 (PMC13016293; doi:10.1371/journal.pone.0341135)

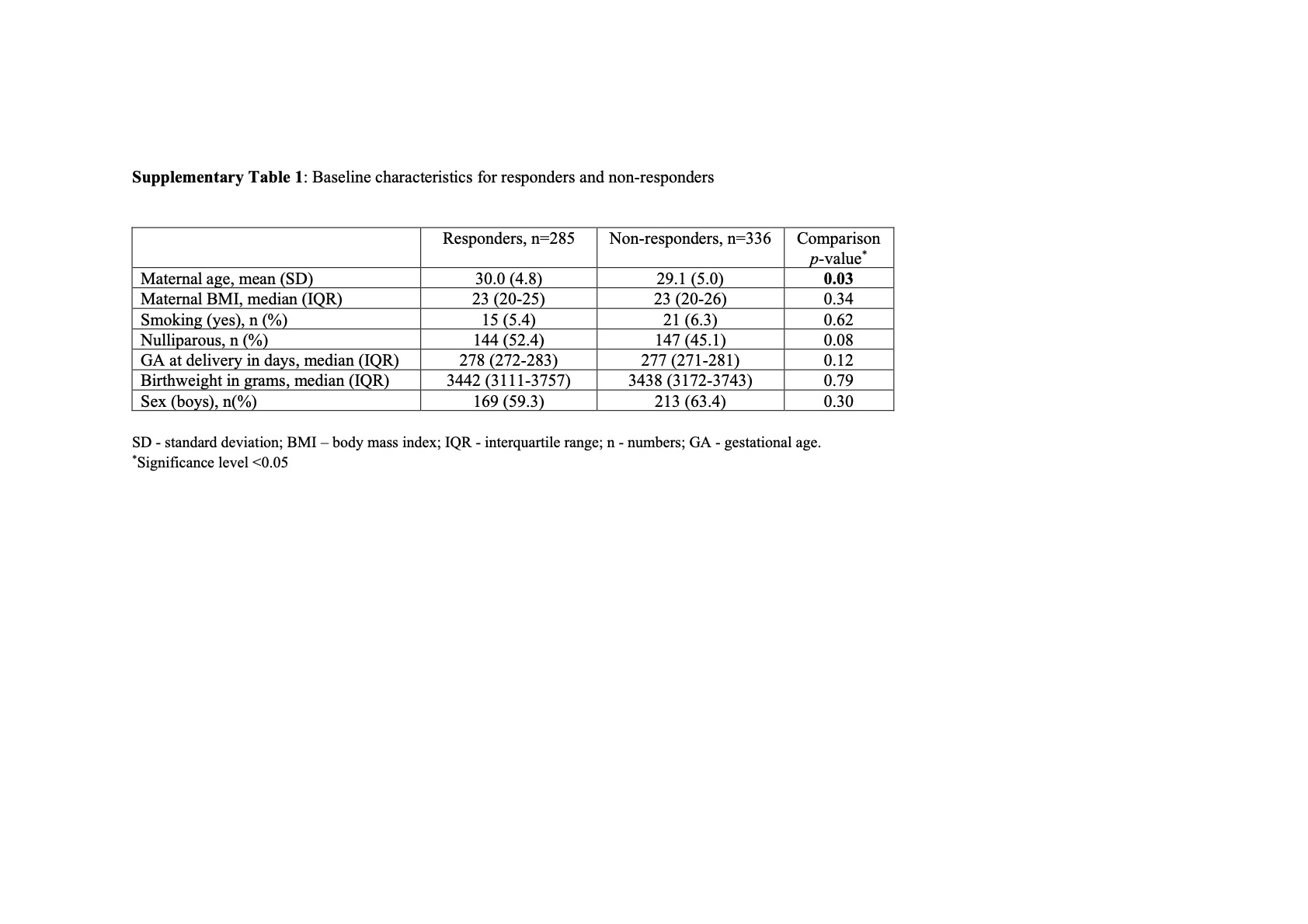

Supplement: S1 Table — SD – standard deviation; BMI – body mass index; IQR – interquartile range; n – numbers; GA – gestational age. *Significance level <0.05. (JPG) [file pone.0341135.s001.jpg]
