# Supplementary material for: Neurodevelopmental outcomes in preschool children with congenital heart defects: A case-control study using Ages & Stages Questionnaire
Source: PLoS One. 2026 Mar 25;21(3):e0341135. doi: 10.1371/journal.pone.0341135 (PMC13016293; doi:10.1371/journal.pone.0341135)
Supplement: Attachment — Submitted filename: Response to Reviewers.pdf [file pone.0341135.s003.pdf]

Rigshospitalet

Dear Editor and reviewers,

We would like to thank you for your comments and suggestions aimed at improving our manuscript, "Neurodevelopmental Outcomes in Preschool Children with Congenital Heart Defects: A Case-Control Study using Ages&Stages Questionnaire".

We sincerely appreciate the time and effort invested providing constructive feedback. We have carefully addressed all points raised, as detailed below. Please do not hesitate to contact us should any clarifications or further revisions be required.

Comments are in bold, and changes made to the manuscript are highlighted with track changes.

We hope you will find the revised manuscript acceptable for *PLOS ONE*.

Sincerely and on behalf of the co-authors,

Simone Hansen, MD

Reviewer 1:

The manuscript addresses an important and clinically relevant topic: neurodevelopmental outcomes in preschool children with surgically corrected congenital heart defects (CHD), assessed with the Ages & Stages Questionnaire (ASQ). The study is well-designed, based on nationwide data, and uses appropriate methodology. My comments are as following:

1) The exclusion criteria may have inadvertently omitted critical cases, particularly those admitted to the neonatal ICU. Moreover, the outcomes may also depend on the surgical success rate; please provide more detailed information on this aspect.

Thank you for this comment. We acknowledge that the exclusion of the most severe CHD cases may have influenced our findings. In particular, children with univentricular heart disease (UVH), who are known to have a higher risk of adverse neurodevelopmental outcomes, were not included. As now clarified in the Discussion, this reflects the very small number of survivors eligible for population-based follow-up in Denmark, due to high prenatal detection (>99%), frequent termination of pregnancy, and substantial postnatal mortality. These factors may help explain the comparable ASQ scores observed between cases and controls. We have updated the Discussion section of the manuscript accordingly (page 13, lines 265-275).

Regarding surgical success, all included children underwent corrective surgery according to current national standards in a centralized setting. Nationwide data indicate that overall postnatal mortality for major CHD is below 10%, with approximately half of deaths occurring in infants who were not considered for or scheduled to undergo surgery. Mortality among CHD subgroups included in the present study was low, supporting that our cohort largely represents surgically treated survivors. We hope that this adequately addresses the reviewer´s concern, and we would be happy to provide further clarification if needed.

2) The methodology related to the use of the ASQ requires further clarification and justification, as the current description is not sufficient.

We thank the reviewer for this comment, which has helped improve the manuscript. We have revised the ASQ-related methodology by moving and rephrasing the relevant content from the Discussion section to the Methods section, also in response to Reviewer 2’s concern. This has resulted in a more complete description of the methodology, and we hope that it adequately addresses the reviewer’s request for clarification (pages 6-7, lines 147-155).

Rigshospitalet

3) The follow-up period is relatively short, which limits the ability to assess long-term neurodevelopmental outcomes.

Thank you for this comment, which was also noted by Reviewer 2. We agree that the follow-up period is relatively short and limits the assessment of long-term neurodevelopmental outcomes. Developmental assessments in early childhood have only a moderate ability to predict later cognitive function. Accordingly, we have clarified in the Discussion that the present study reflects outcomes limited to the preschool period, without formal assessment of cognitive function or academic achievement. We further note that future studies with consecutive follow-up, potentially of the same cohort, will be needed to assess long-term neurodevelopmental outcomes in children with surgically corrected CHD (page 16, lines 342-347).

Reviewer 2:

Congratulations on the good job.
1. Abstract
i. Excellent summary
ii. Proper inference
2. Framing – disclosures, funding, and ethical issues
a. Excellent
3. Introduction, methods, results, and conclusion

a. The response rate amongst controls is 36.5%. Such may mean skewed result. Kindly relook the impact on the results.

Thank you for this comment. Although the response rate among controls was 36.5%, the observed effect size was small (Cohen's d = 0.23), indicating limited impact on the overall results. This limitation has been acknowledged in the Discussion (page 16, lines 332-341).

b. Discussion section shouldn't take a lot of explanations to convince readers the methodology was proper. You better remove it and can drop some points at the methods section if you wish.

We agree that the Discussion should not be used to justify the methodology. Accordingly, we have removed this content from the Discussion section and relocated the relevant methodological details to the Methods section. This revision also aligns well with Reviewer 1's comment requesting further clarification of ASQ-related methodology (pages 6-7, lines 147-155).

c. Prevalence of congenital heart diseases and screening isn't a target and doesn't need discussion. Was better finalized on the introduction. It took a content here.

We agree and have substantially shortened and revised this section in the Discussion, sharpening the focus on potential explanations for the comparable ASQ scores between cases and controls, rather than providing a general description of prenatal detection of major CHD and the screening program (page 13, lines 261-265). Relevant background information has been retained in the Methods section.

d. The subgroup analysis had created minimal sample sizes, which remained to be a limitation.

We agree that the subgroup analyses resulted in small sample sizes, which constitutes a limitation. This is acknowledged in the Discussion section (page 15, lines 329-330).

e. Long – term neurodevelopmental outcomes

We thank the reviewer for this helpful comment, which was also noted by Reviewer 1. We agree that the follow-up period is relatively short and limits the assessment of long-term neurodevelopmental outcomes. Developmental assessments in early childhood have only a moderate ability to predict later cognitive function. Accordingly, we have clarified in the Discussion that the present study reflects outcomes limited to the preschool period, without formal assessment of cognitive function or academic achievement. We further note that future studies with consecutive follow-up, potentially of the same cohort, will be needed to assess long-term neurodevelopmental outcomes in children with surgically corrected CHD (page 16, lines 342-347).

f. Term of astonishment like surprisingly better is avoided.

We appreciate this comment. We have revised the manuscript accordingly by removing the term "surprisingly" from the two relevant sentences (page 12, lines 257-258, and page 16, lines 351-352).
